# Bioprosthetic vs. Mechanical Mitral Valve Replacement for Rheumatic Heart Disease in Patients Aged 50–70 Years

**DOI:** 10.3389/fcvm.2022.904958

**Published:** 2022-05-31

**Authors:** Jun Yu, Wei Wang

**Affiliations:** Department of Structural Heart Disease Centre, Fu Wai Hospital, National Center for Cardiovascular Diseases, Chinese Academy of Medical Sciences and Peking Union Medical College, Beijing, China

**Keywords:** bioprosthesis, rheumatic, mechanical valve, mitral valve replacement, heart valve diseases

## Abstract

**Background:**

Rheumatic heart disease (RHD) is a critical problem in developing countries and is the cause of most of the cardiovascular adverse events in young people. In patients aged 50–70 years with RHD requiring mitral valve replacement (MVR), deciding between bioprosthetic and mechanical prosthetic valves remains controversial because few studies have defined the long-term outcomes.

**Methods:**

1,691 Patients aged 50–70 years with RHD who received mechanical mitral valve replacement (MVRm) or bioprosthetic mitral valve replacement (MVRb) were retrospectively reviewed in Fuwai hospital from 2010 to 2014. Follow-up ended 31/12/2021; median duration was 8.0 years [interquartile range (IQR), 7.7–8.3 years]. Propensity score matching at a 1:1 ratio for 24 baseline features between MVRm and MVRb yielded 300 patient pairs. The primary late outcome was postoperative mid- to long-term all-cause mortality.

**Results:**

Ten-year survival after MVR was 63.4% in the MVRm group and 63.7% in the MVRb group (HR, 0.91; 95% CI, 0.69–1.21; *P* = 0.528). The cumulative incidence of mitral valve reoperation was 0.0% in the MVRm group and 1.2% in the MVRb group (HR, 0.92; 95% CI, 0.69–1.21; *P* = 0.530). The cumulative incidence of stroke was 5.5% in the MVRm group and 6.1% in the MVRb group (HR, 0.89; 95% CI, 0.67–1.18; *P* = 0.430). The cumulative incidence of major bleeding events was 3.3% in the MVRm group and 3.4% in the MVRb group (HR, 0.92; 95% CI, 0.70–1.22; *P* = 0.560).

**Conclusions:**

In patients aged 50–70 years with RHD who underwent mitral valve replacement, there was no significant difference on survival, stroke, mitral valve reoperation and major bleeding events at 10 years. These findings suggest mechanical mitral valve replacement may be a more reasonable alternative in patients aged 50–70 years with rheumatic heart disease.

## Introduction

Rheumatic heart disease (RHD) remains a challenging health problem across the worldwide, especially in developing countries and is a major cause of cardiovascular mortality in young people (1).

Rheumatic heart disease is mainly caused by rheumatic fever. Rheumatic fever is a type of recurrent acute or chronic systemic connective tissue inflammation caused by group a beta-hemolytic streptococcus invasion of genetically susceptible people (2). After an acute attack, heart damage of varying severity is often left; especially valvular disease is the most dangerous, resulting in chronic rheumatic heart disease or rheumatic heart valve disease (3). The mitral valve is most commonly involved in clinical practice. For rheumatic mitral valve disease, the main treatment methods are surgical operations, including mitral valvuloplasty and mitral valve replacement. At present, the best surgical approach for rheumatic mitral valve disease is still controversial, but due to the higher risk of reoperation with mitral valvuloplasty, mitral valve replacement is more commonly used in clinical practice (4).

The artificial valve used in mitral valve replacement can be categorized as mechanical valve and biological valve. Both types of prosthetic valves have advantages and disadvantages. Patients using mechanical valves need to take anticoagulants for life, and are prone to complications such as premature ventricular contractions, thromboembolism, and bleeding (5, 6). However, mechanical valves have better durability and are less likely to undergo secondary surgery. Patients using biological valve do not need long-term anticoagulation, which reduces the risk of bleeding and embolism, but is more prone to structural valve deterioration (SVD) and has a higher risk of mitral valve reoperation. A mechanical valve may be an option when the patient is already on anticoagulation therapy or when the risk of reoperation is high. When patients have poor compliance with anticoagulation therapy, or lack corresponding medical conditions to monitor coagulation index, bioprosthetic valves can be considered. The trade-off between bleeding risk and reoperation is critical and involves many factors. However, age becomes one of the most objective factors in choosing the appropriate valve type (7). For patients requiring mitral valve replacement, the European Society of Cardiology (ESC) recommends a mechanical valve for those under 65 years of age and a biological valve for those over 70 years of age (5). The American Heart Association (AHA) recommends the use of mechanical valves for people under the age of 50, and the use of biological valves for people over 70 years old, and it indicates that uncertainty and debate continue about which type of prosthesis is appropriate for patients 50–70 years of age. There are conflicting data on survival benefit of mechanical vs. bioprosthetic valves in this age group (6). Few studies have explicitly compared mechanical valves and bioprosthetic valves in patients with RHD (8–10). Therefore, current guidelines do not provide a choice of the most appropriate valve type for patients aged 50–70 years with rheumatic heart disease.

Generally, younger patients may be more inclined to use a mechanical mitral valve, but with the development of transcatheter mitral valve replacement, the problem of reoperation after bioprosthetic valve deterioration may not be so difficult. The rise of this technology may also have important implications for the choice of valve type (7). Therefore, we conducted this study to compare long-term survival and incidence of related outcomes for patients aged 50–70 years with rheumatic mitral valve disease.

## Methods

### Study Design

All patients aged 50–70 years with RHD who underwent primary mitral valve replacement in Fuwai hospital from 01/01/2010 to 31/12/2014 were identified for retrospective cohort study. The Medical Ethics Review Committee of Fuwai Hospital approved this study (No. 2,021–1,545). Informed consent was waived.

Mechanical prosthetic and bioprosthetic valve replacements were differentiated using International Classification of Diseases, Ninth Revision, Clinical Modification (ICD-9-CM) procedure codes 35.23 and 35.24, respectively. The following patients were excluded: (I) patients who had undergone prior replacement of any heart valve; (II) patients who had undergone concomitant replacement of the aortic, pulmonary, or tricuspid valves; repair of the aortic or pulmonary valves; (III) concomitant coronary artery bypass graft surgery; or concomitant thoracic aortic surgery.

To minimize potential selection bias, we calculated a propensity score from selected variables and matched each patient in the bioprosthetic group with each patient in the mechanical prosthetic group. Three hundred patient pairs of the mechanical prosthetic group and bioprosthetic group were identified and were eligible for analysis.

### Outcomes

The primary outcomes of this study were all-cause mortality. In-hospital or 30-day outcomes included all-cause mortality, stroke, major bleeding events, acute kidney injury, respiratory failure, heart failure, readmission, reexploration for bleeding and deep wound infection. Late outcomes included stroke, mitral valve reoperation, thromboembolic events, major bleeding events, infective endocarditis, prosthetic valve endocarditis, readmission for heart failure, and all-cause readmission. Stroke was defined as any cerebrovascular accident documented during the index hospitalization as well as any subsequent hospital admission in which the principal diagnosis was hemorrhagic or ischemic stroke (not including transient ischemic attacks). Reoperation was defined as any subsequent MVR. Major bleeding event was defined as requiring hospitalization or blood transfusion. Patients were censored on 31/12/2021.

### Statistical Analysis

Baseline patient characteristics are represented as means with standard deviations for normally distributed continuous variables, medians and interquartile ranges for non-normal distributed continuous variables, and proportions for categorical variables. Shapiro-Wilk test was used to test normal distribution. To compare baseline differences in comorbidity between patients receiving mechanical prosthetic and bioprosthetic valves, the Student *t*-test or Mann-Whitey *U*-test was performed for continuous variables, the Pearson χ2 test or Fisher's exact test was performed for categorical variables, and standardized differences were calculated for all variables.

Confounding due to differences in baseline characteristics was addressed using propensity score matching. To calculate the propensity score, a hierarchical logistic regression model was fitted with bioprosthetic implantation as the outcome. Covariates entered into the model include all measured baseline characteristics: age, sex, year of surgery, New York Heart Association (NYHA) class III to IV, admission urgency, hypertension, diabetes mellitus, hyperlipidaemia, choronic kidney disease, active endocarditis or sepsis, chronic obstructive pulmonary disease, cerebrovascular disease, peripheral vascular disease, coronary artery disease, congestive heart failure, atrial fibrillation, other arrhythmia, liver disease, history of cancer, history of mitral balloon dilatation, mean pulmonary artery systolic blood pressure (greater than or equal to 50 mmHg), concomitant tricuspid valve repair, concomitant radiofrequency ablation of atrial fibrillation, concomitant thrombectomy.

The area under the receiver operating characteristic curve for this model was 0.79. A 1:1 match was then performed using a caliper of 0.4 of the logit of the propensity score computed by this model. The baseline characteristics of the patient pairs matched by propensity score were compared using the paired *t-*test or the Wilcoxon Signed-Rank Test for continuous variables and the McNemar test for categorical variables. Standardized difference that was <0.1 was deemed indicative of acceptable balance. In-hospital or 30-day outcomes rates were compared using the McNemar test.

For the primary end point, survival curves and 10-year estimates were derived from Kaplan-Meier method. For the secondary end points of other time to event outcomes, a competing risk analysis was performed to construct cumulative incidence function curves and to calculate 10-year estimates. For all end points, marginal Cox proportional hazards regression models with robust sandwich variance estimators were fitted with only prosthesis type entered as a covariate. The difference in overall survival was compared using the Cox model, whereas the differences in secondary end points were evaluated using the Gray test. All tests were 2-tailed with an α level of 0.05. All statistical analyses were performed using R software version 4.1.0.

## Results

### Patient Characteristics

**Figure 1:**
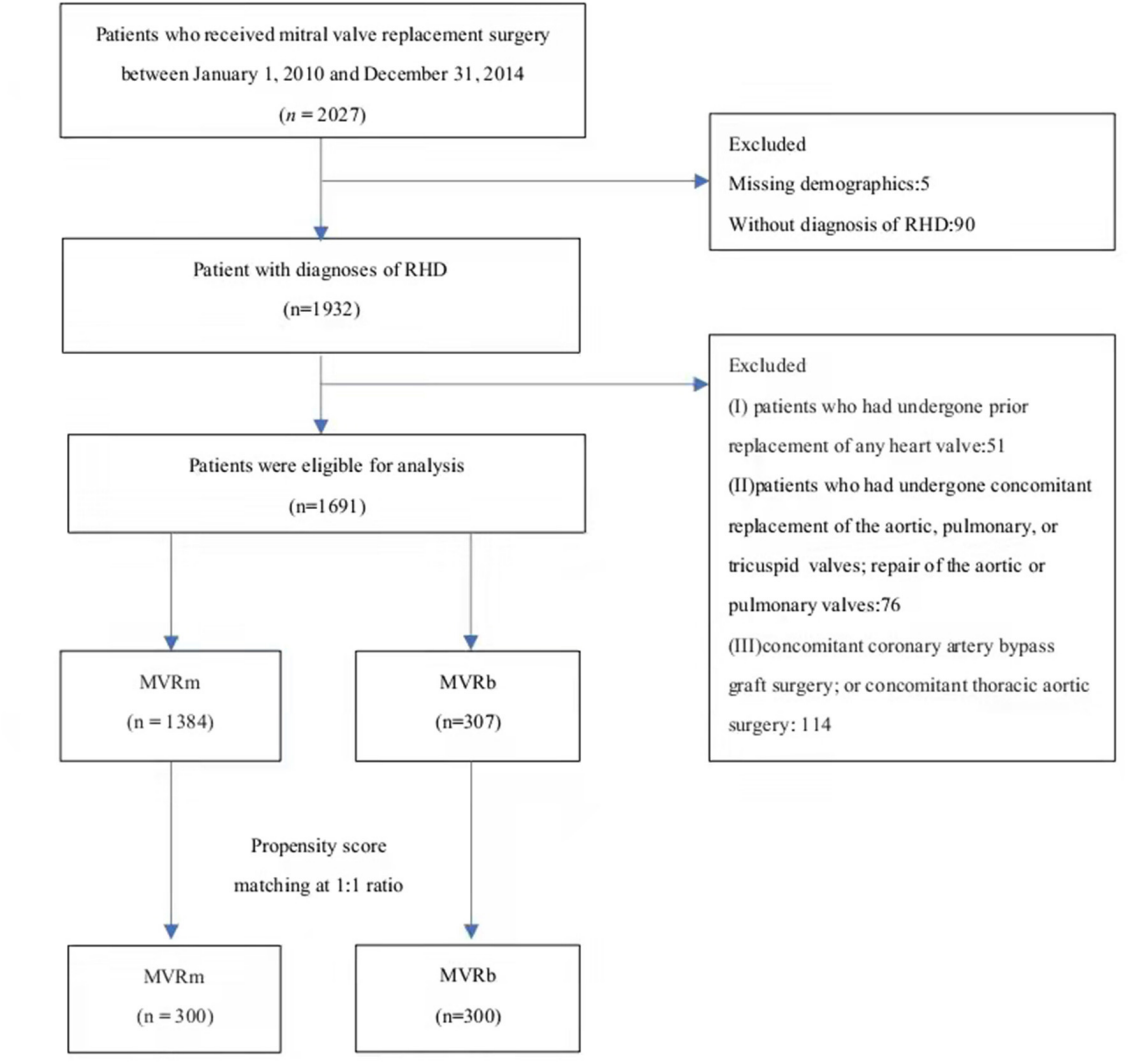
The flowchart of patient inclusion and exclusion. RHD, Rheumatic heart disease. MVRm, mechanical mitral valve replacement; MVRb, bioprosthetic mitral valve replacement.

Between January 2010 and December 2014, 2,027 patients who underwent MVR were included in this study, among whom 1,932 were diagnosed as having RHD and 1,691 were eligible for inclusion. Among the included patients, 1,384 (81.8%) selected a mechanical valve and 307 (18.2%) selected a bioprosthetic valve (Figure 1). The baseline characteristics of the overall cohort are presented in Table 1. Patients who received bioprosthetic valve replacement (*n* = 307) compared those who received mechanical valve replacement (*n* = 1,384) were older (61.9 ± 4.9 vs. 57.1 ± 4.8 years, *P* < 0.001), and more likely to have a history of hypertension (22.5 vs. 15.7%, *P* = 005), diabetes mellitus (11.1 vs. 7.1%, *P* = 0.025), NYHA class III-IV (35.2 vs. 29.0, *P* = 040). Patients who received mechanical prosthetic valves were more likely to have cardiovascular morbidity including severe pulmonary hypertension (16.1 vs. 10.1%, *P* = 010), atrial fibrillation (73.4 vs. 65.6%, *P* = 0.006). But patients in MVRb groups were more likely to receive concomitant atrial fibrillation radiofrequency ablation (26.4 vs. 18.0%, *P* = 001). A ratio of 1:1 propensity-score matching produced 300 patient pairs. Age and all baseline comorbidities were balanced with the two groups (Table 2).

**Table 1:** Patient baseline characteristics in the overall cohort according to type of mitral valve replacement.

	**All patients** **(*n* = 1,691)**	**MVRm** **(*n* = 1,384)**	**MVRb** **(*n* = 307)**	**SMD, %**	***P*** **-value**
Age, [mean (SD)], y	58.0 (5.1)	57.1 (4.8)	61.9 (4.9)	98.8	<0.001
Male sex	398 (23.5)	319 (23.0)	79 (25.7)	6.1	0.353
NYHA class III-IV	510 (30.2)	402 (29.0)	108 (35.2)	12.8	0.040
Emergent or urgent admission status	339 (20.0)	286 (20.7)	53 (17.3)	9.0	0.205
History of mitral valve ballon dilation	101 (6.0)	82 (5.9)	19 (6.2)	1.1	0.965
Hypertension	286 (16.9)	217 (15.7)	69 (22.5)	16.3	0.005
Diabetes mellitus	132 (7.8)	98 (7.1)	34 (11.1)	12.7	0.025
Hyperlipidaemia	311 (18.4)	240 (17.3)	71 (23.1)	13.7	0.022
Endocarditis or sepsis	2 (0.1)	2 (0.1)	0 (0.0)	4.2	1.000
Chronic obstructive pulmonary disease	17 (1.0)	12 (0.9)	5 (1.6)	6.0	0.371
Peripheral vascular disease	39 (2.3)	35 (2.5)	4 (1.3)	10.8	0.278
Cerebrovascular disease	226 (13.4)	178 (12.9)	48 (15.6)	7.6	0.230
Heart failure	4 (0.2)	3 (0.2)	1 (0.3)	1.9	1.000
Chronic kidney disease	4 (0.2)	3 (0.2)	1 (0.3)	1.9	1.000
Liver disease	17 (1.0)	15 (1.1)	2 (0.7)	5.4	0.711
Cancer	6 (0.4)	5 (0.4)	1 (0.3)	0.6	1.000
Pulmonary artery systolic pressure (≥50 mmHg)	254 (15.0)	223 (16.1)	31 (10.1)	20.0	0.010
**Coronary artery disease**					
Without coronary artery disease	1,636 (96.8)	1,336 (96.5)	300 (97.7)		
Without prior revascularization	52 (3.1)	45 (3.3)	7 (2.3)	9.4	0.479
Prior PCI	3 (0.2)	3 (0.2)	0 (0.0)		
**Arrhythmia**					
Atrial fibrillation	1,217 (72.0)	1,016 (73.4)	201 (65.5)	16.7	0.006
Other type of arrhythmia	103 (6.1)	83 (6.0)	20 (6.5)	2.1	0.833
**Concomitant procedures**					
Tricuspid valve repair	1,300 (76.9)	1,071 (77.4)	229 (74.6)	6.4	0.330
Atrial fibrillation radiofrequency ablation	330 (19.5)	249 (18.0)	81 (26.4)	19.0	0.001
Atrial thrombectomy	328 (19.4)	270 (19.5)	58 (18.9)	1.6	0.867
**Year of surgery**					
2010	272 (16.1)	209 (15.1)	63 (20.5)		
2011	314 (18.6)	266 (19.2)	48 (15.6)		
2012	285 (16.9)	255 (18.4)	30 (9.8)	9.8	0.111
2013	414 (24.5)	362 (26.2)	52 (16.9)		
2014	406 (24.0)	292 (21.1)	114 (37.1)		

*Data are given as n (%) except where otherwise noted. MVRm, mechanical mitral valve replacement; MVRb, bioprosthetic mitral valve replacement; SMD, standardized mean difference; PCI, percutaneous coronary intervention; NYHA, New York Heart Association*.

**Table 2:** Baseline characteristics after propensity score matching.

	**MVRm** **(*n* = 300)**	**MVRb** **(*n* = 300)**	**SMD, %**	***P*** **-value**
Age, [mean (SD)], y	61.60 (4.58)	61.78 (4.82)	3.8	0.627
Male sex	73 (24.3)	77 (25.7)	3.1	0.777
NYHA class III-IV	100 (33.3)	106 (35.3)	4.2	0.667
Emergent or urgent admission status	51 (17.0)	52 (17.3)	0.9	1.000
History of mitral valve ballon dilation	25 (8.3)	19 (6.3)	8.3	0.434
Hypertension	62 (20.7)	65 (21.7)	2.4	0.842
Diabetes mellitus	27 (9.0)	32 (10.7)	5.3	0.583
Hyperlipidaemia	79 (26.3)	67 (22.3)	9.5	0.295
Endocarditis or sepsis	0 (0.0)	0 (0.0)	0.0	1.000
Chronic obstructive pulmonary disease	4 (1.3)	5 (1.7)	2.6	1.000
Peripheral vascular disease	3 (1.0)	4 (1.3)	2.9	1.000
Cerebrovascular disease	53 (17.7)	47 (15.7)	5.5	0.584
Heart failure	1 (0.3)	1 (0.3)	0.0	1.000
Chronic kidney disease	2 (0.7)	1 (0.3)	5.9	1.000
Liver disease	2 (0.7)	2 (0.7)	0.0	1.000
Cancer	0 (0.0)	1 (0.3)	5.9	1.000
Pulmonary artery systolic pressure (≥50 mmHg)	30 (10.0)	30 (10.0)	0.0	1.000
**Coronary artery disease**				
Without coronary artery disease	294 (98.0)	293 (97.7)		
Without prior revascularization	5 (1.7)	7 (2.3)	0.0	0.513
Prior PCI	1 (0.3)	0 (0.0)		
**Arrhythmia**				
Atrial fibrillation	208 (69.3)	198 (66.0)	7.0	0.432
Other type of arrhythmia	14 (4.7)	20 (6.7)	8.1	0.377
**Concomitant procedures**				
Tricuspid valve repair	233 (77.7)	224 (74.7)	6.9	0.443
Atrial fibrillation radiofrequency ablation	80 (26.7)	78 (26.0)	1.5	0.926
Atrial thrombectomy	62 (20.7)	56 (18.7)	5.1	0.608
**Year of surgery**				
2010	36 (12.0)	61 (20.3)		
2011	55 (18.3)	48 (16.0)	0.4	0.956
2012	56 (18.7)	29 (9.7)		
2013	78 (26.0)	51 (17.0)		
2014	75 (25.0)	111 (37.0)		

*Data are given as n (%) except where otherwise noted. MVRm, mechanical mitral valve replacement; MVRb, bioprosthetic mitral valve replacement; SMD, standardized mean difference; PCI, percutaneous coronary intervention; NYHA, New York Heart Association*.

### In-hospital or 30-Day Outcomes

Among patients matched by propensity score, there was no significant difference in 30-day mortality (0.3% in the bioprosthesis group vs. 0.3% in the mechanical prosthesis group, *P* = 1.000) after valve replacement. The incidence of 30-day complications and outcomes was comparable between the 2 groups after matching (Table 3).

**Table 3:** In-hospital or 30-day outcomes of mitral valve replacement in patients matched by propensity score.

	**MVRm** **(*n* = 300)**	**MVRb** **(*n* = 300)**	***P*** **-value**
Mortality	1 (0.3)	1 (0.3)	1.000
Stroke	2 (0.7)	0 (0.0)	0.249
Major bleeding events	2 (0.7)	0 (0.0)	0.499
Acute kidney injury	2 (0.7)	0 (0.0)	0.499
Respiratory failure	3 (1.0)	0 (0.0)	0.249
Heart failure	3 (1.0)	0 (0.0)	0.249
Readmission	2 (0.7)	2 (0.7)	1.000
Re-exploration for bleeding	5 (1.7)	3 (1.0)	0.725
Deep wound infection	3 (1.0)	3 (1.0)	1.000

*Data are given as n (%) except where otherwise noted. MVRm, mechanical mitral valve replacement; MVRb, bioprosthetic mitral valve replacement*.

### Late Outcomes

#### Survival

**Figure 2:**
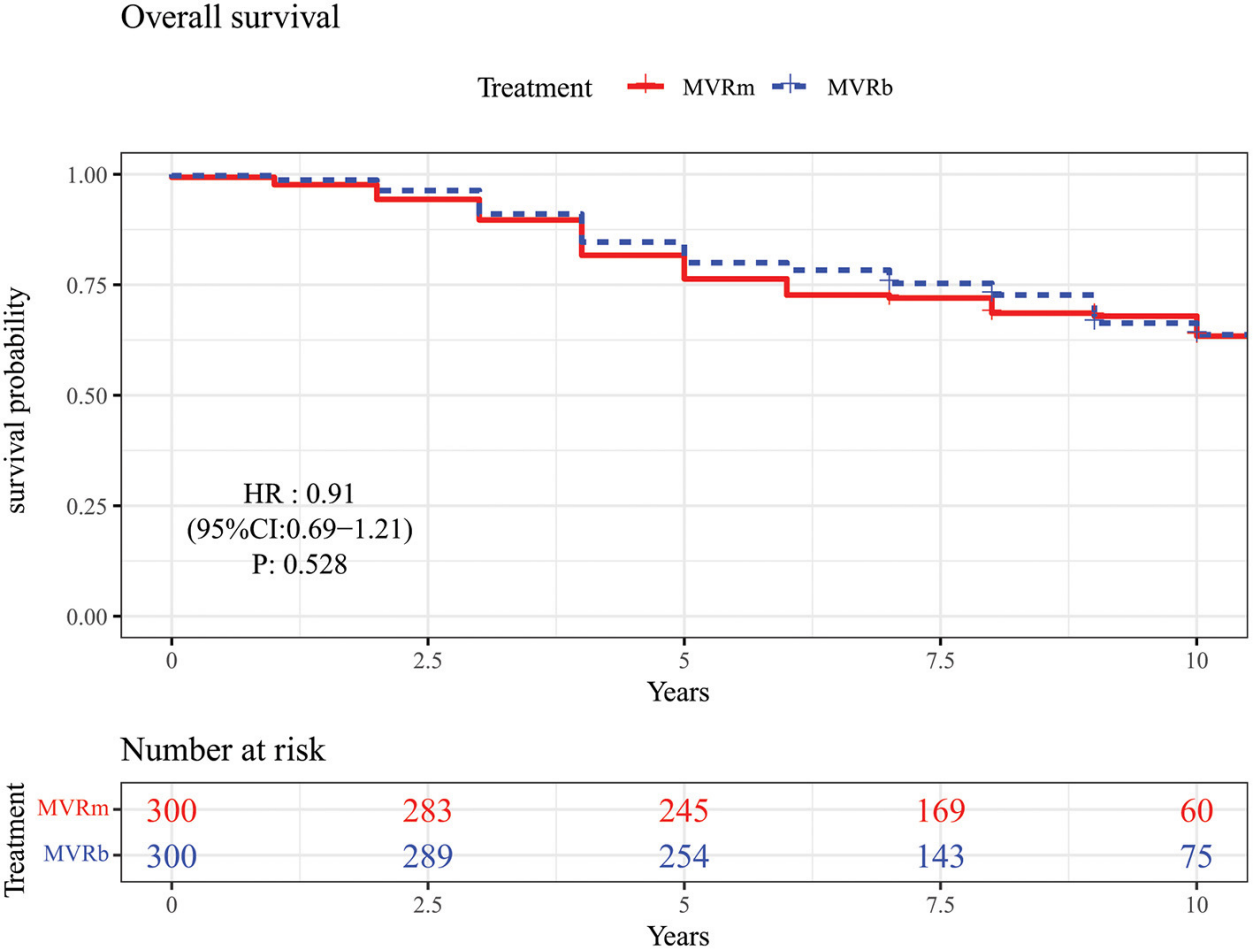
Overall survival in patients aged 50–70 years after mitral valve replacement according to prosthetic type. MVRm, mechanical mitral valve replacement; MVRb, bioprosthetic mitral valve replacement.

Among patients matched by propensity score, there was no significant difference in mid- to long-term survival between MVRm and MVRb [hazard ratio (HR), 0.91 (95% CI, 0.69–1.21), *P* = 0.528; Figure 2]. A total of 98 (32.7%) death occurred in the MVRm group and 92 (30.7%) deaths occurred in the MVRb group. The actuarial survival at 1, 3, 5, and 10 years were 97.7, 89.7, 76.3, and 63.4% in the MVRm group, and 98.7, 91.0, 80.0, and 63.7% in the MVRb group, respectively.

### Mitral Valve Reoperation

**Figure 3:**
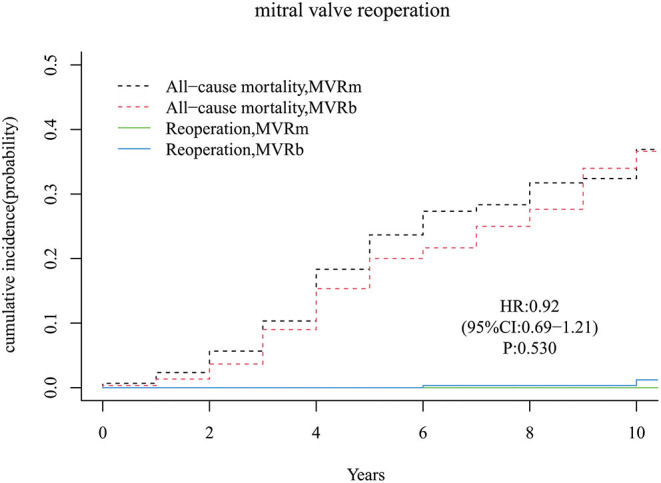
Cumulative incidence of reoperation. MVRm, mechanical mitral valve replacement; MVRb, bioprosthetic mitral valve replacement.

There were only 3 patients received a mitral valve reoperation in the MVRb and none in the MVRm. But the difference of the cumulative incidence was not significant [hazard ratio (HR), 0.92 (95% CI, 0.69–1.21), *P* = 0.530; Figure 3] between the 2 groups. The cumulative incidence of mitral valve reoperations at 3, 5, 7, and 10 years were 0.0, 0.0, 0.0, and 0.0% in the MVRm, and 0.0, 0.0, 0.3, and 1.2% in the MVRb, respectively.

### Infective Endocarditis

**Figure 4:**
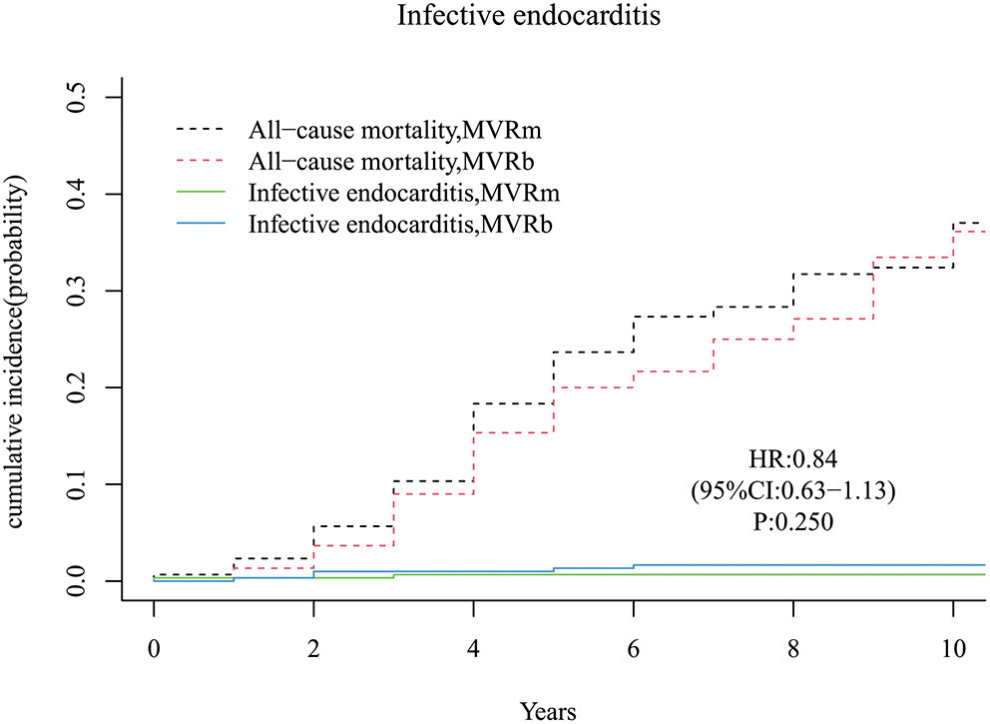
Cumulative incidence of infective endocarditis. MVRm, mechanical mitral valve replacement; MVRb, bioprosthetic mitral valve replacement.

A total of 2 infective endocarditis occurred in the MVRm and 5 infective endocarditis occurred in the MVRb. There was no significant difference in cumulative incidence of infective endocarditis between the MVRm and the MVRb [hazard ratio (HR), 0.84 (95% CI, 0.63–1.13), *P* = 250; Figure 4]. The cumulative incidence of infective endocarditis at 3, 5, 7, and 10 years were 0.7, 0.7, 0.7, and 0.7% in the MVRm, and 1.0, 1.3, 1.7, and 1.7% in the MVRb, respectively.

### Stroke

**Figure 5:**
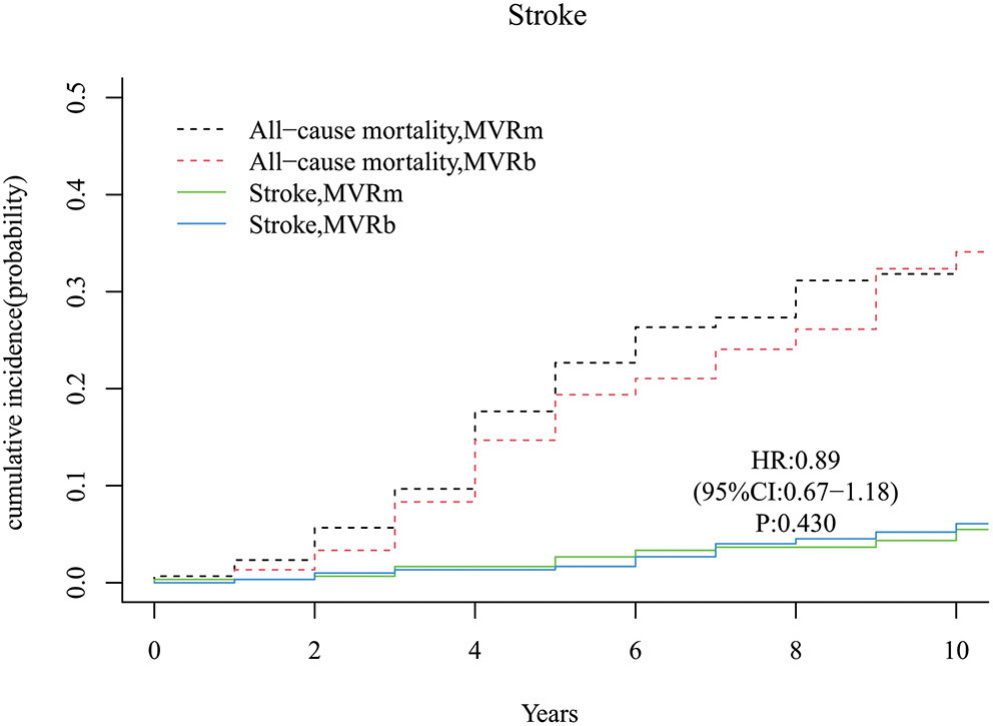
Cumulative incidence of stroke. MVRm, mechanical mitral valve replacement; MVRb, bioprosthetic mitral valve replacement.

A total of 28 strokes occurred during follow-up time, 13 in the MVRm group and 15 in the MVRb group. The cumulative incidence of stroke after MVR was no significant difference between the MVRm and the MVRb [hazard ratio (HR), 0.89 (95% CI, 0.67–1.18), *P* = 430; Figure 5]. Among the 28 patients of stroke, 9 were hemorrhagic and 19 were ischemic. Of the 19 ischemic strokes, 9 occurred in the MVRm group and 10 occurred in the MVRb group.

### Readmission for Heart Failure

**Figure 6:**
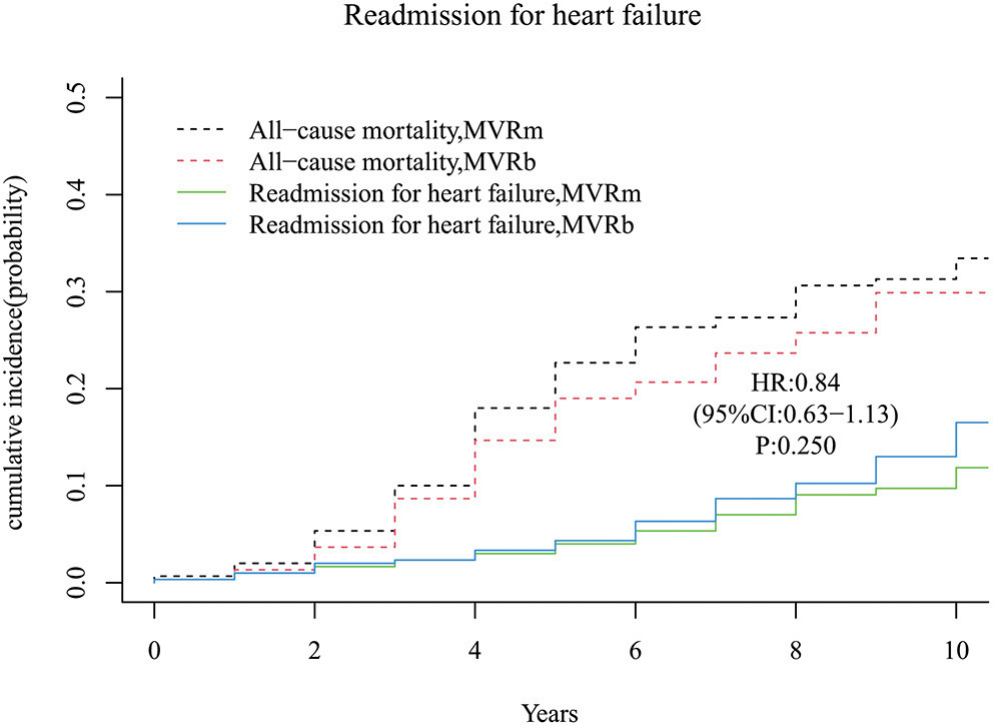
Cumulative incidence of readmission for heart failure. MVRm, mechanical mitral valve replacement; MVRb, bioprosthetic mitral valve replacement.

A total of 31 and 38 patients occurred readmission for heart failure in the MVRm group and in the MVRb group, respectively. There was no significant difference of readmission for heart failure between the 2 groups [hazard ratio (HR), 0.84 (95% CI, 0.63–1.13), *P* = 250; Figure 6]. The cumulative incidence of readmission for heart failure at 3, 5, 7, and 10 years were 2.3, 4.0, 7.0, and 11.9% in the MVRm group, and 2.3, 4.3, 8.7, and 16.5% in the MVRb group, respectively.

### Readmission for Any Cause

**Figure 7:**
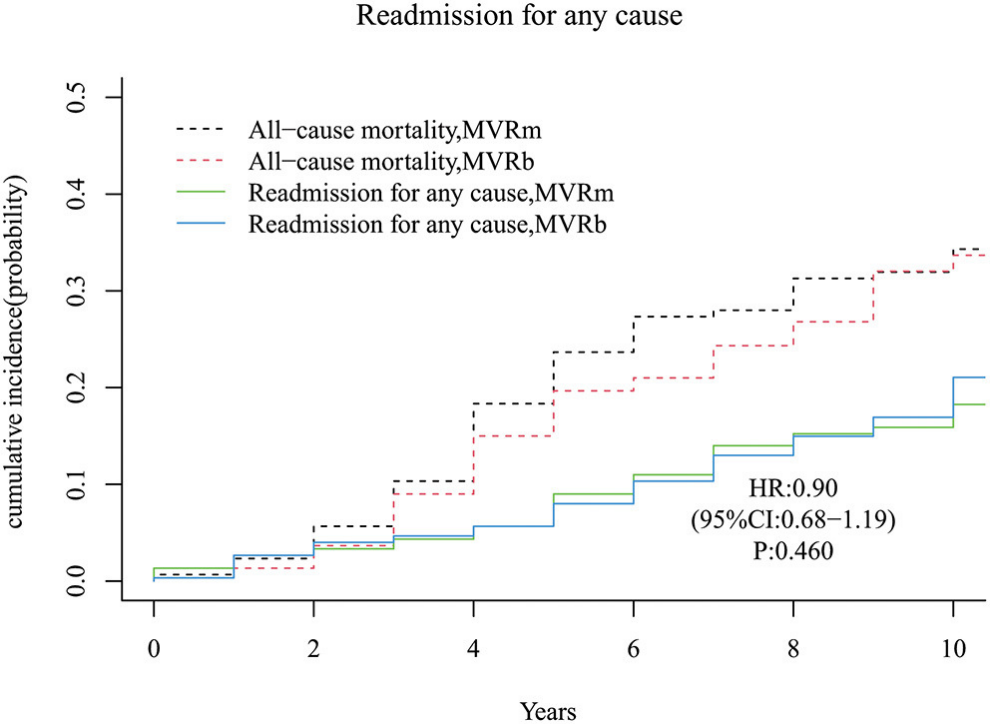
Cumulative incidence of readmission for any cause. MVRm, mechanical mitral valve replacement; MVRb, bioprosthetic mitral valve replacement.

Forty-nine patients occurred readmission for any cause in the MVRm group while 52 patients occurred readmission for any cause in the MVRb group during follow-up period. The cumulative incidence of readmission for any cause after MVR was no significant difference between the MVRm and the MVRb [Hazard Ratio (HR), 0.90 (95% CI, 0.68–1.19), *P* = 460; Figure 7]. The cumulative incidence of readmission for any cause at 3, 5, 7, and 10 years were 4.3, 9.0, 14.0, and 18.3% in the MVRm, and 4.7, 8.0, 13.0, and 21.1% in the MVRb, respectively.

### Thromboembolic Events

**Figure 8:**
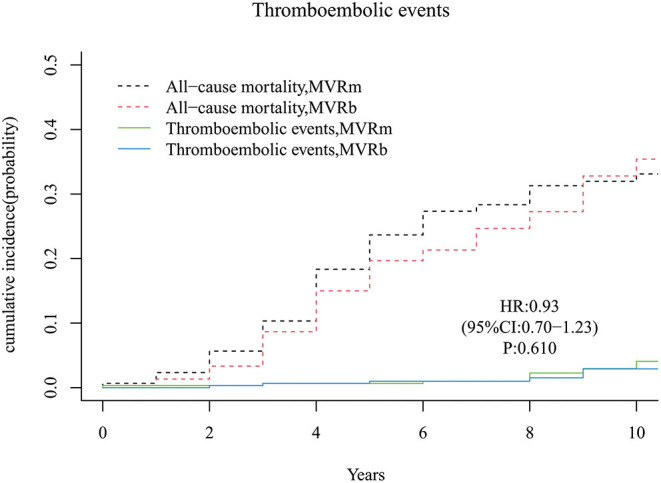
Cumulative incidence of thromboembolic events. MVRm, mechanical mitral valve replacement; MVRb, bioprosthetic mitral valve replacement.

A total of 14 thromboembolic events occurred during follow-up period, 8 in the MVRm group and 6 in the MVRb group. No significant difference was observed between the MVRm and the MVRb [hazard ratio (HR), 0.93 (95% CI, 0.70–1.23), *P* = 610; Figure 8]. The cumulative incidence of thromboembolic events at 3, 5, 7 and 10 years were 0.7, 0.7, 1.0, and 4.1% in the MVRm, and 0.7, 1.0, 1.0, and 2.9% in the MVRb, respectively. Among the 14 patients of thromboembolic events, 6 were acute myocardial infarction (5 in the MVRm vs. 1 in the MVRb), 2 were bowel ischemia (1 in the MVRm vs. 1 in the MVRb), 3 were pulmonary embolism (1 in the MVRm vs. 2 in the MVRb) and 3 were systemic thromboembolism (3 in the MVRm vs. 0 in the MVRb).

### Major Bleeding Events

**Figure 9:**
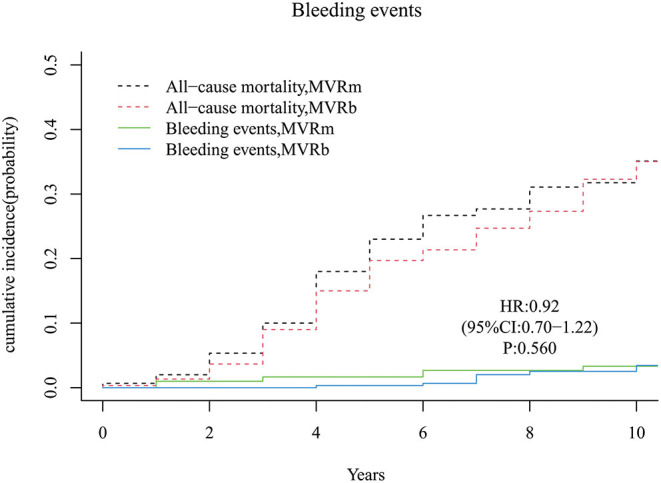
Cumulative incidence of major bleeding events. MVRm, mechanical mitral valve replacement; MVRb, bioprosthetic mitral valve replacement.

There was no significant difference of major bleeding events in patients with a mechanical or a biological prosthesis [hazard ratio (HR), 0.92 (95% CI, 0.70–1.22), *P* = 560; Figure 9]. The cumulative incidence of major bleeding events at 3, 5, 7, and 10 years were 1.7, 1.7, 2.7, 3.3% in the MVRm, and 0.0, 0.3, 2.0, and 3.4% in the MVRb, respectively. Major bleeding events (10 in the MVRm vs. 8 in the MVRb) were most commonly intracerebral hemorrhage (8 in the MVRm vs. 7 in the MVRb). Of all major bleeding events, 2 were gastrointestinal bleedings (1 in the MVRm vs. 1 in the MVRb).

### Moderate or Severe Perivalvular Leakage

**Figure 10:**
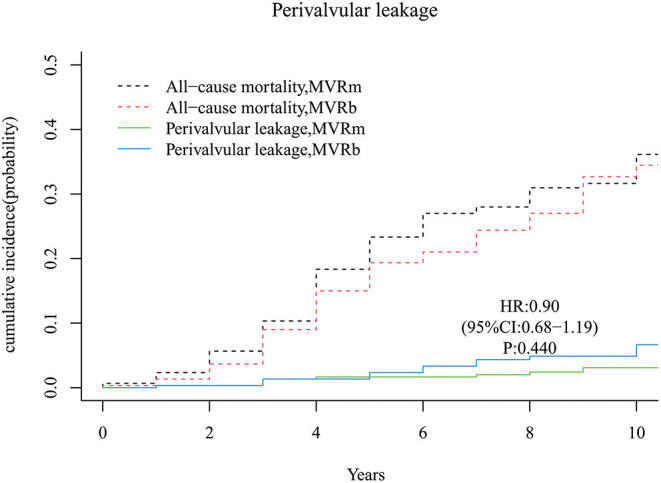
Cumulative incidence of perivalvular leakage. MVRm, mechanical mitral valve replacement; MVRb, bioprosthetic mitral valve replacement.

A total of 8 moderate or severe perivalvular leakage occurred in the MVRm and 17 perivalvular leakage occurred in the MVRb during follow up. There was no significant difference between the 2 groups [hazard ratio (HR), 0.90 (95% CI, 0.68–1.19), *P* = 440; Figure 10]. The cumulative incidence of moderate or severe perivalvular leakage at 3, 5, 7, and 10 years were 1.3, 1.7, 2.0, and 3.1% in the MVRm, and 1.3, 2.3, 4.4, and 6.7% in the MVRb, respectively.

## Discussion

There is still much controversy about which type of valve to choose for patients aged 50–70 who need mitral valve replacement in current clinical practice (11). Current guidelines also fail to decide the best options for patients in this age group (6). When we provide advice to patients, we generally consider factors such as age, life expectancy, reoperation risk, anticoagulation related events, and patient preference. However, the specific etiology of valvular disease is rarely considered. Different etiologies of heart valve disease, such as rheumatic, degenerative, and infectious (12), may also affect the clinical prognosis. The study by Goldstone et al. (13) showed that the long-term mortality benefit that was associated with a mechanical prosthesis, as compared with a biologic prosthesis, persisted until 70 years of age among patients undergoing mitral valve replacement. However, Chikwe et al. (14) demonstrates that there was no significant difference in survival at 15-year follow-up between mechanical prosthetic and bioprosthetic mitral valve replacements. Both of the two large studies were conducted in the United States, and neither classified the specific etiology of valvular heart disease. The difference in the etiological composition of valvular heart disease may be one of the underlying reasons for the wide disparity in the results of the two studies. Kulik et al. (15) found that there was no significant difference in late mortality in MVR patients aged 50–65, but an increase in the requirement for reoperation for bioprosthetic valves and an increased risk of thromboembolism for mechanical valves. However, the study did not analyze the specific etiology of valvular heart disease. A study analyzed patients aged 50–70 with infective endocarditis for mitral valve replacement, and the results showed that the long-term mortality and reoperation rates of the biological valve group were significantly higher than those of the mechanical valve group. There were no significant differences in stroke and major bleeding events (16). A retrospective study of patients with RHD from Taiwan showed that the all-cause mortality and reoperation rates in the biological valve group were higher than those in the mechanical valve group, no group differences were observed in the risks of stroke, thromboembolic events, and major bleeding events (7). In our study, in patients with RHD aged 50–70 years, there was no significant difference in all-cause mortality and reoperation rates between the MVRb group and the MVRm group. Therefore, whether the specific etiology of valvular heart disease will affect the long-term clinical outcomes after mitral valve replacement requires further clinical trials and newer high-quality evidence.

Our study showed that the bioprosthetic valve utilization rate in our hospital increased from 23.2% in 2010 to 28.1% in 2014. This trend is similar to that of the United States (17). With the increasing use of bioprostheses, the clinical prognosis of patients using bioprostheses has become an issue that we need to pay more attention to. The results of this study showed that there was no significant difference between the bioprosthetic valve group and the mechanical valve group in both primary and secondary outcomes for patients aged 50–70 years with RHD, indicating the use of bioprosthetic valves seems to be a good choice, too. After all, long-term anticoagulation is not required, and the quality of life could be improved. But the follow-up is unfortunately too short to assess the durability of the bioprostheses, that won't be enough time to develop SVD. Mitral valve reoperation rate was higher in MVRb group although the difference was not significant. Our study showed that the mechanical valve group did not increase the incidence of stroke, major bleeding and thromboembolic events compared with the bioprosthetic valve group even if the MVRm group need anticoagulation, suggesting that the use of mechanical valve in patients aged 50–70 years with rheumatic mitral valve disease is a better choice, especially for patients with atrial fibrillation. In this study, 72.0% patients who underwent MVR had atrial fibrillation. And mechanical valve is generally considered more durable than biological valve, reducing the risk of reoperation. During the next years will be quite sure that the MVRb group starts to develop SVD, and the advantage of the MVRm group taking over. With the advent of transcatheter mitral valve technology, some studies have reported that valve-in-valve procedures are associated with better outcomes compared with valve-in-ring procedures (18–20). For high-risk patients who need secondary surgery after SVD, the technology of transcatheter mitral valve replacement can significantly reduce the risk compared with conventional open-heart surgery, providing these patients with a better opportunity to replace the valve, which is of great significance (18). However, we recommend mechanical prostheses in this patient group for the first operation because transcatheter mitral valve-in-valve replacement would not be better in terms of mortality, rehospitalization, and cost-effectiveness, particularly. Besides, with the advancement of science and technology, monitoring INR is more convenient (21, 22). Therefore, the trend of bioprostheses toward younger patients should be tempered according to our study.

### Study Limitations

The main limitation was the nature of the single-center retrospective study. Although we used propensity score matching to minimize measured confounders, potential confounding variables not measured could not be adjusted in this study. There may not have been adequate control for selection bias. The 10-year follow-up was insufficient to fully assess lifetime risks, particularly of SVD and reoperation. However, there were no significant differences in 30-day mortality and morbidity in this cohort, suggesting that the treatment groups were well-matched. Finally, the relatively large sample size and complete follow-up in our study can be considered precise and trustworthy.

## Conclusions

This propensity score-matched study compared clinical outcomes between mechanical and bioprosthetic MVR in patients aged 50–70 years with rheumatic heart disease. Despite the trend of bioprostheses toward younger patients, mechanical mitral valve replacement may be a more reasonable alternative in this patient group without an increased risk of stroke or major bleeding events.

## Perspective Statement

Either a mechanical or bioprosthetic valve is used in patients undergoing mitral valve replacement (MVR). As the development of the transcatheter intervention technologies, the use of bioprosthesis increased during the past decades. But we found that mechanical prosthesis may be a more reasonable alternative in patients aged 50–70 years with rheumatic heart disease.

## Data Availability Statement

The raw data supporting the conclusions of this article will be made available by the authors, without undue reservation.

## Ethics Statement

The studies involving human participants were reviewed and approved by the Medical Ethics Review Committee of Fuwai Hospital approved this study (No. 2021–1545). Written informed consent for participation was not required for this study in accordance with the national legislation and the institutional requirements.

## Author Contributions

JY and WW contributed to conception and design of the study. JY organized the database, performed the statistical analysis, and wrote the first draft of the manuscript. All authors contributed to manuscript revision, read, and approved the submitted version.

## Funding

This work was supported by the project of clinical trials in Fuwai Hospital (grant number: 2021-HG34, Beijing, China).

## Conflict of Interest

The authors declare that the research was conducted in the absence of any commercial or financial relationships that could be construed as a potential conflict of interest.

## Publisher's Note

All claims expressed in this article are solely those of the authors and do not necessarily represent those of their affiliated organizations, or those of the publisher, the editors and the reviewers. Any product that may be evaluated in this article, or claim that may be made by its manufacturer, is not guaranteed or endorsed by the publisher.
